# Biglycan-driven risk stratification in ZFTA-RELA fusion supratentorial ependymomas through transcriptome profiling

**DOI:** 10.1186/s40478-024-01921-w

**Published:** 2025-01-07

**Authors:** Konstantin Okonechnikov, David R. Ghasemi, Daniel Schrimpf, Svenja Tonn, Martin Mynarek, Jan Koster, Till Milde, Tuyu Zheng, Philipp Sievers, Felix Sahm, David T.W. Jones, Andreas von Deimling, Stefan M. Pfister, Marcel Kool, Kristian W. Pajtler, Andrey Korshunov

**Affiliations:** 1 Hopp Children’s Cancer Center Heidelberg (KiTZ), Heidelberg, Germany; 2 Division of Pediatric Neuro-Oncology (B062), German Cancer Research Center (DKFZ) and German Cancer Consortium (DKTK), Heidelberg, Germany; 3 Clinical Cooperation Unit Neuropathology (B300), German Cancer Research Center (DKFZ), German Cancer Consortium (DKTK), National Center for Tumor Diseases (NCT), Heidelberg, Germany; 4 Department of Neuropathology, Heidelberg University Hospital, Heidelberg, Germany; 5 Pediatric Hematology and Oncology, University Medical Center Hamburg-Eppendorf, Hamburg, Germany; 6 Mildred Scheel Cancer Career Center HaTriCS4, University Medical Center Hamburg-Eppendorf, Hamburg, Germany; 7 Research Institute Children’s Cancer Center, Hamburg, Germany; 8 Center for Experimental and Molecular Medicine, Amsterdam University Medical Centers, University of Amsterdam and Cancer Center Amsterdam, Amsterdam, The Netherlands; 9 Clinical Cooperation Unit Pediatric Oncology, German Cancer Research Center (DKFZ) and German Consortium for Translational Cancer Research (DKTK), Heidelberg, Germany; 10 Department of Pediatric Hematology and Oncology, Heidelberg University Hospital, Heidelberg, Germany; 11 National Center for Tumor Diseases (NCT), Heidelberg, Germany; 12 Division of Pediatric Glioma Research (B360), German Cancer Research Center (DKFZ), Heidelberg, Germany; 13 Princess Máxima Center for Pediatric Oncology, Utrecht, 3584CS The Netherlands; 14 Clinical Cooperation Unit Neuropathology (B300), German Cancer Research Center (DKFZ), Im Neuenheimer Feld 280, 69120 Heidelberg, Germany

**Keywords:** Ependymoma, *ZFTA-RELA* fusion, *BGN*, Expression, Prognosis

## Abstract

Recent genomic studies have allowed the subdivision of intracranial ependymomas into molecularly distinct groups with highly specific clinical features and outcomes. The majority of supratentorial ependymomas (ST-EPN) harbor *ZFTA-RELA* fusions which were designated, in general, as an intermediate risk tumor variant. However, molecular prognosticators within ST-EPN ZFTA-RELA have not been determined yet. Here, we performed methylation-based DNA profiling and transcriptome RNA sequencing analysis of 80 ST-EPN ZFTA-RELA investigating the clinical significance of various molecular patterns. The principal types of *ZFTA-RELA* fusions, based on breakpoint location, demonstrated no significant correlations with clinical outcomes. Multigene analysis disclosed 1892 survival-associated genes, and a metagene set of 100 genes subdivided ST-EPN ZFTA-RELA into favorable and unfavorable transcriptome subtypes composed of different cell subpopulations as detected by deconvolution analysis. *BGN* (biglycan) was identified as the top-ranked survival-associated gene and high *BGN* expression levels were associated with poor survival (Hazard Ratio 17.85 for PFS and 45.48 for OS; log-rank; p-value < 0.01). Furthermore, BGN immunopositivity was identified as a strong prognostic indicator of poor survival in ST-EPN, and this finding was confirmed in an independent validation set of 56 samples. Our results indicate that integrating *BGN* expression (at mRNA and/or protein level) into risk stratification models may improve ST-EPN ZFTA-RELA outcome prediction. Therefore, gene and/or protein expression analyses for this molecular marker could be adopted for ST-EPN ZFTA-RELA prognostication and may help assign patients to optimal therapies in prospective clinical trials.

**Supplementary Information:**

The online version contains supplementary material available at 10.1186/s40478-024-01921-w.

## Introduction

Ependymomas (EPN) are neuroepithelial malignancies of the central nervous system (CNS), accounting for 5% of all CNS tumors in children. The mainstay of treatment for EPN remains surgery and radiotherapy (RT), whereas chemotherapy (CHT) is currently not a consistent component of standard-of-care protocols [17, 27, 30, 32, 35].

Recent genomic studies enabled the subdivision of supratentorial (ST), posterior fossa (PF), and spinal (SP) EPN into molecularly distinct groups with variable clinical features and outcomes. Within the ST CNS compartment, underlying molecular signatures including DNA methylation and transcriptome analysis define three major subgroups, designated as ST-subependymoma (ST-SE; 5-year overall survival – 100%), ST-EPN YAP1 (5-year overall survival – 100%), and ST-EPN RELA (5-year overall survival – 75–80%) [1, 24, 27, 28, 30]. The latest version of the WHO classification of CNS tumors includes two molecularly defined types of ST-EPN: *ZFTA* fusion-positive and *YAP1* fusion-positive [33].

The vast majority of these tumors (ca. 85%) designated as ST-EPN ZFTA-RELA, contain oncogenic fusions between *ZFTA*, a transcriptional activator harboring zinc finger domains, and *RELA*, the principal effector of canonical NFκB signaling [2, 19, 23, 27, 29, 37]. The *ZFTA–RELA* fusion is sufficient to drive tumor formation in vivo due to active proliferation of neural stem cells in the cerebral cortex [2, 19, 37]. In addition, some infrequent ST-EPN harbor *ZFTA* fusions to gene partners other than *RELA* such as *MAML2/MAML3*, *NCOA1/NCOA2*, and others [37, 38].

Despite of the detailed genomic characterization of ST-EPN ZFTA-RELA, robust molecular prognosticators determining the clinical course of these ependymal neoplasms with variable outcomes have not been determined yet [5, 14, 17, 18, 28, 37]. The objective of the current study was to identify prognostically tractable molecular marker(s) to elaborate on an optimal risk stratification of ST-EPN ZFTA-RELA, suitable for application in routine clinical settings. For these purposes, we performed an integrative RNA-based analysis of a representative ST-EPN ZFTA-RELA cohort with sustained patients’ follow-up also accompanied with additional data types including DNA methylation and IHC profiling.

## Materials and methods

### The patient population of molecularly diagnosed ST-EPN with ZFTA-RELA fusion

A cohort of 80 CNS tumors diagnosed as ST-EPN ZFTA-RELA with DNA methylation profiling (see below) was selected from the previously published international EPN set that was molecularly analyzed at the German Cancer Research Center [28, 37]. Informed consent was obtained from all patients’ parents or other relatives/caregivers. This retrospective study was conducted under the auspices of the local Ethics Committees in adherence to the tenets of the Declaration of Helsinki.

All 80 samples were classified as “EPN_ST_ZFTA_RELA” using the MNP2.0 v12.5 Random Forest classifier (www.molecularneuropathology.org) with a calibrated prediction score > 0.90. Identification of the molecular group was confirmed using t-distributed stochastic neighbor embedding (t-SNE) and uniform manifold approximation and projection for dimension reduction (UMAP) methods, as described [37]. Differential methylation analysis was performed via minfi R package [27]. Treatment details and follow-up data were available for all patients. The follow-up analysis was stalled on 01.01.2024 as the end-point, with a median observation time of 92 months. Progression-free survival (PFS) was calculated from the date of diagnosis until tumor recurrence or last contact for disease-free patients. Overall survival (OS) was calculated from the date of diagnosis until the death of a patient from disease or last contact for patients who were still alive.

### RNA sequencing analysis

RNA was extracted from formalin-fixed and paraffin-embedded (FFPE) tissue samples and RNA sequencing was performed on a NextSeq 500 or NovaSeq 6000 instruments (Illumina) as described [31]. The reads were aligned to hg38 reference using STAR version 2.5.2b [9] and for each sample, gene expression was quantified by the feature counts module of the Subread package version 1.4.6 [20] using Gencode version 38 annotations with uniquely mapped reads only. Fusion discovery was conducted based on RNA sequencing data using two independent algorithms: InFusion v0.6.3 [25] and Arriba v1.2.0 [34] with standard parameters as described previously [37]. Reverse-transcriptase (RT)-PCR was also used to validate the presence of fusion transcripts in 58 cases.

Tumor sample comparison was based on the selection of the top most variable genes with *log2* RPKM expression normalization. Differential gene expression analysis between various tumor groups was performed by comparing one molecular class against the other using Limma package (adjusted p-value < 0.05). Gene ontology analysis was done using ClueGO with visualization [4] using Cytoscape version 3.4. Additional visualization and analyses were performed using R2: Genomics Analysis and Visualization Platform (http://r2.amc.nl). Multiple gene survival analysis was performed with R2 survival package using a cut-off in expression that resulted in the highest and lowest log-rank p-value using a Bonferroni correction for multiple testing. For the development of transcriptome-based risk stratification for ST-EPN ZFTA-RELA, a combination of survival-associated genes (or metagene set) with an optimal log-rank p-value for OS was identified with R2 as a k-mean supervised clustering applying standard parameters (transformation – log2 Z-score; floor value – 16; the number of passes − 10). Gene set enrichment analysis (GSEA) for identified transcriptome subtypes was also performed with R2.

Deconvolution analysis was performed with the BayesPrism tool [6] using the raw gene expression count matrices of the bulk dataset and of the corresponding EPN single-cell RNA-seq dataset [14] as the reference to impute the fractions of the single-cell populations. Statistical evidence of a relative difference in cell type proportions between prognostically relevant ST-EPN transcriptome subtypes was measured with a t-test, afterwards applying Benjamini-Hochberg correction per subgroup with a limit cut-off for an adjusted p-value of 0.05. To verify the deconvolution results, gene set variance analysis (GSVA) [15] was performed on mean gene expression values computed from normalized matrices for target EPN SGS sample cohort with distinction on favorable/unfavorable sample sets. The target gene lists for each cell type were obtained from the corresponding study [14].

### Immunohistochemistry (IHC) with biglycan antibody

IHC was conducted on 4-µm thick FFPE tissue sections mounted on adhesive slides followed by drying at 80 °C for 15 min. For IHC analysis, a rabbit monoclonal biglycan antibody (PA5-72823, Abcam) was applied. IHC was performed with an automated immunostainer (Benchmark; Ventana XT) using antigen-retrieval protocol CC1 and a working antibody dilution of 1:1000 for 2 with incubation at 37 °C for 32 min. IHC with EMA, L1CAM, p65-RelA antibodies was performed as described previously [12, 23, 26].

### Statistics

The distributions of PFS and OS were calculated according to the Kaplan-Meier method using the log-rank test. For multivariate analysis, Cox proportional hazards regression models were used and estimated hazard ratios are provided with 95% confidence intervals. The ability of Cox models to classify risk was assessed by computing the area under the time-dependent receiver operating characteristic (ROC) curves, calculated according to the Nearest Neighbor Estimation (NNE) method. ROC curves were computed every 18 months of follow-up time up to 10 years, and the resulting areas under the curve were compared by paired t-test. Statistical analyses were performed with R 3.5.1, with packages “survival’, “survminer” and “maxstat” for uni and multivariate survival analyses, “pec” and “survivalROC” for prediction error and ROC curves.

### Data availability

The RNA-seq dataset generated and analyzed in the current study (normalized gene expression counts matrix) with detailed annotation is available in the R2 platform (http://r2.amc.nl) under the name “Tumor Ependymoma FFPE - Korshunov − 80 - RPKM - epffpe”. The methylation data available in GEO database under access number GSE65362.

## Results

### Clinical and pathological characteristics of ST-EPN ZFTA-RELA

**Fig. 1:**
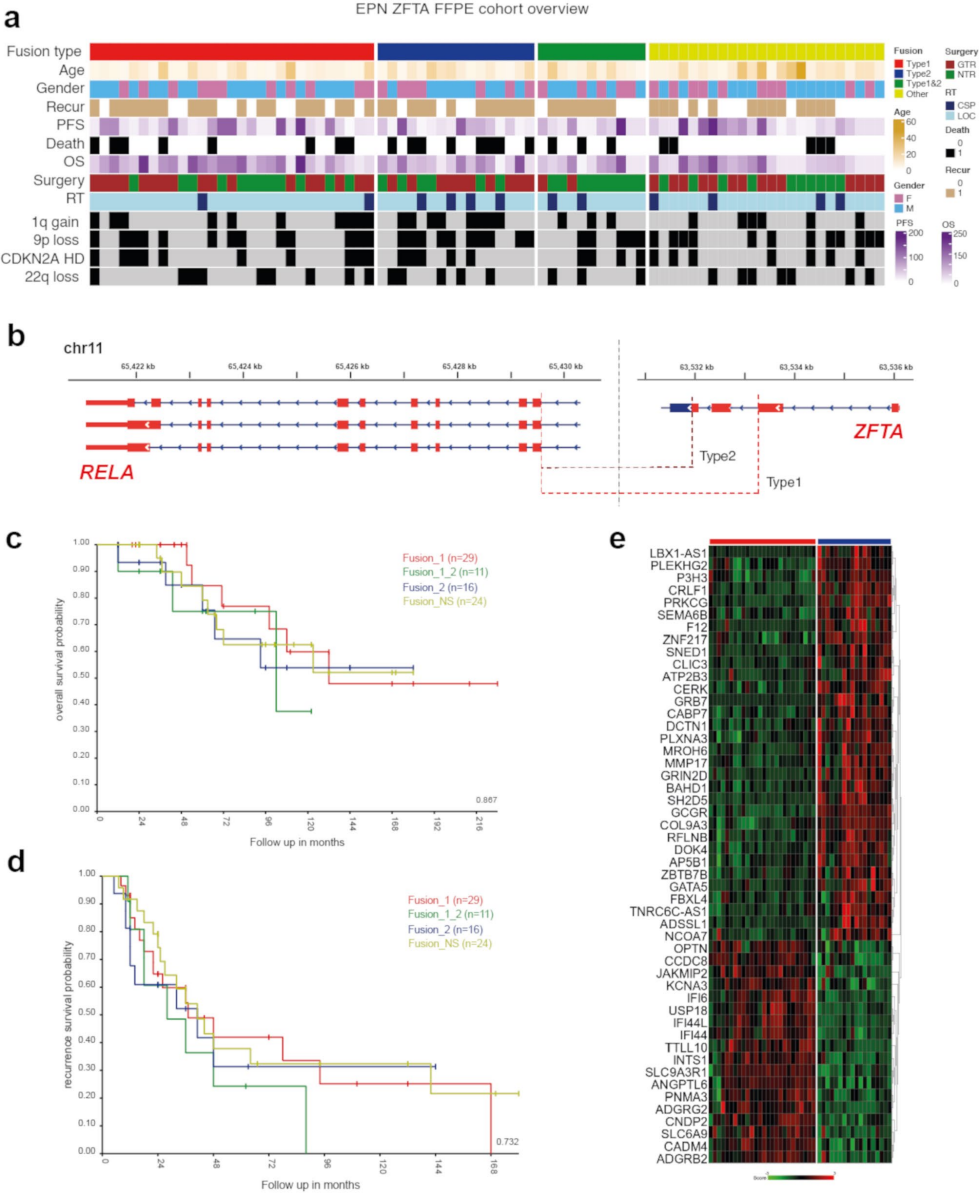
**a**) Annotation onco-plot describing patient histological and molecular characteristics for target ZFTA-RELA ST-EPN tumors with available RNA sequencing data (*n* = 80). The following abbreviations were used: RT - radiotherapy, LOC - conformal local, CSP - craniospinal, PFS—progression-free survival, CNV—copy number variants. **b**) Genomic locations the ZFTA-RELA fusion breakpoints stating the main types of the fusion. **c**, **d**) No survival differences were identified between the various ZFTA-RELA fusion types. **d**) Heatmap of significant differentially expressed genes between ZFTA-RELA fusion type 1 (*n* = 29) and 2 (*n* = 16)

The clinical and molecular characteristics of 80 patients with ST-EPN ZFTA-RELA are summarized in Fig. 1a; Table 1 and Suppl. Table 1. Patients were aged between 4 and 64 years (median: 11.3), with a preponderance of patients younger than 18 years (85% vs. 15% adult patients), and a male: female ratio of 2.5:1. Only a minority of patients (4%) were diagnosed as M2-3 stages at initial presentation. All 80 patients were treated with maximal safe surgical resection and received postoperative radiotherapy (RT), either conformal local RT in 68 patients (85%) or craniospinal RT in 12 patients (15%). Fifty-eight patients (65%) received chemotherapy (HIT-based protocol) after RT. Tumor histology was identified as anaplastic EPN (EPN Grade 3). Dot-like EMA, membranous L1CAM, and nuclear p65-RelA expression were identified in all samples analyzed. Disease relapses occurred in 47 of the patients (60%) and 41 relapsed patients (88%) were treated with second-line surgery, re-irradiation (either conformal or radiosurgery), and/or chemotherapy with various regimens. Twenty-two (28%) of relapsed patients succumbed to disease, 33 patients (41%) showed “no evidence of disease” at last follow-up, and 25 patients (31%) were “alive with disease”. Recurrent copy number variants (CNVs) observed in > 20% of cases were 1q gain (35%), 9p loss (50%) accompanied with 9p21/*CDKN2A/B* homozygous deletion in 30%, 22q loss (30%), and monosomy X (30%). In line with previous retrospective studies [27, 30], 5-year PFS was 45%, 5-year OS was 82%, and 10-year OS was 61% for this cohort, and no clinical or cytogenetic variables were associated with patients’ survival (Table 2).

**Table 1:** Clinical and molecular variables for ST-EPN ZFTA-RELA with various fusion variants

Variable	Type 1 (29/35%)	Type 2 (16/20%)	Type 3 (11/15%)	Type 4 (24/30%)
Age: Median; Child/Adult	10; 75%/25%	11; 85%/15%	12; 85%/15%	11; 75%/25%
Gender: Male/Female	60%/40%	50%/50%	65%/35%	65%/35%
M stage: M0 vs. M2-3	95%/5%	100%/0	90%/10%	95%/5%
Removal: Gross total/Near total	50%/50%	60%/40%	50%/50%	50%/50%
Radiotherapy: Local vs. CSI	90%/10%	85%/15%	100%/0	90%/10%
Chemotherapy	60%	65%	65%	65%
5-year PFS	45%	40%	35%	45%
5-year OS	95%	85%	75%	80%
1q gain	35%	50%	25%	25%
9p loss	45%	60%	40%	45%
*CDKN2A/B* homozygous deletion	30%	30%	30%	30%
22q loss	30%	30%	30%	30%
Monosomy X	35%	30%	30%	35%

**Table 2:** Uni- and multivariate overall survival analyses for ZFTA-RELA-fused ST-EPN cohort

Variables	HRU* PFS	P-Val	HRU OS	P-Val	HRM** PFS	P-Val	HRM OS	P-Val
Age: (children vs. adult)	1.17	0.48	1.48	0.22	1.27	0.61	3.23	0.11
Gender (male vs. female)	1.25	0.26	0.59	0.44	1.29	0.41	1.21	0.71
Removal (Gross total vs. Near total)	0.08	0.74	0.12	0.71	0.55	0.09	0.91	0.87
Chemotherapy (yes vs. no)	0.12	0.34	0.18	0.14	0.24	0.46	0.56	0.77
1q gain (yes vs. no)	2.18	0.18	2.44	0.11	0.83	0.68	0.61	0.44
9p loss (yes vs. no)	2.43	0.12	1.26	0.26	1.39	0.48	0.71	0.69
*CDKN2A/B* homo deletion (yes vs. no)	2.71	0.11	0.76	0.38	1.44	0.44	1.14	0.91
22q loss (yes. vs. no)	1.68	0.19	2.46	0.11	0.50	0.18	0.38	0.21
Monosomy X (yes vs. no)	1.33	0.22	0.88	0.64	0.77	0.32	0.44	0.38
Fusion type (2 vs. 1)	1.63	0.42	3.11	0.08	0.98	0.95	3.11	0.09
Transcriptome cluster (HR vs. LR)	**14.1**	**< 0.01**	**40.6**	**< 0.01**	**5.12**	**< 0.01**	**10.14**	**< 0.01**
*BGN* expression*** (> 4.0 vs. < 4.0)	**8.82**	**< 0.01**	**23.47**	**< 0.01**	**3.71**	**0.02**	**5.47**	**< 0.01**

* - Hazard ratio univariate; ** - Hazard ratio multivariate; *** - RPKM *log2*

### Types of *ZFTA-RELA* fusions in ST-EPN and their clinical-molecular characteristics

By RNA sequencing, several distinct variants of the *ZFTA-RELA* fusions were identified (Fig. 1b): (i) fusion type 1 – *ZFTA* exons 1_2 and *RELA* exons 2_11 (29/35%); (ii) fusion type 2 – *ZFTA* exons 1_3 and *RELA* exons 2_11 (16/20%); (iii) combined type_1 and type_2 fusions (11/15%), designated as fusion type 3; (iv) other less common *ZFTA-RELA* fusion variants were designated as fusion type 4 (24/30%) (Table 1). Some fusions with involvement of *ZFTA* and other genes were identified in an additional set of ST-EPN cases (*n* = 14) reflecting previously reported results [37], but this subset was excluded from further analysis (data not shown). Among CNVs, 1q gains and losses of 9p were frequent in ST-EPN ZFTA-RELA with fusion type 2 (55% and 60%, respectively). Treatment details were similar for all ST-EPN ZFTA-RELA fusion types and no survival differences were identified between the various fusion variants (Fig. 1c, d).

### Genes differentially expressed between various fusion types of ST-EPN ZFTA-RELA

Comparing transcriptome profiles generated for ST-EPN with *ZFTA-RELA* fusion types 1 (*n* = 29) and 2 (*n* = 16), 134 genes and processed pseudogenes were identified as differentially expressed genes (DEG) between these molecular variants; 98 were overexpressed in ST-EPN ZFTA-RELA with fusion type 1, and 36 in ST-EPN ZFTA-RELA with fusion type 2 (Fig. 1e; Suppl. Table 2). Thus, *INTS1*,* CCDC8*,* ADGRG2*,* KCNA3* were top-ranked genes in ST-EPN ZFTA-RELA type 1, whereas *CRLF1*,* GCGR*,* PRKCG*,* GRIN2D* were the top overexpressed genes for ST-EPN ZFTA-RELA type 2. In turn, transcriptome signatures of ST-EPN ZFTA-RELA type 1 identified with Gene ontology analysis included pathways involved in the cilium/axoneme, immune response, interferon synthesis, response to viral stimulus, and RNA binding. In contrast, signaling pathways identified for ST-EPN ZFTA-RELA type 2 were enriched with genes involved in neuron guidance, tyrosine kinase, transmembrane transport, and phosphorylation (Suppl. Table 3). There were no statistically significant differentially expressed genes identified between ST-EPN ZFTA-RELA with other fusion types, perhaps due to significant molecular variability within ST-EPN fusion groups 3 and 4.

### Gene sets associated with survival ST-EPN ZFTA-RELA

**Fig. 2:**
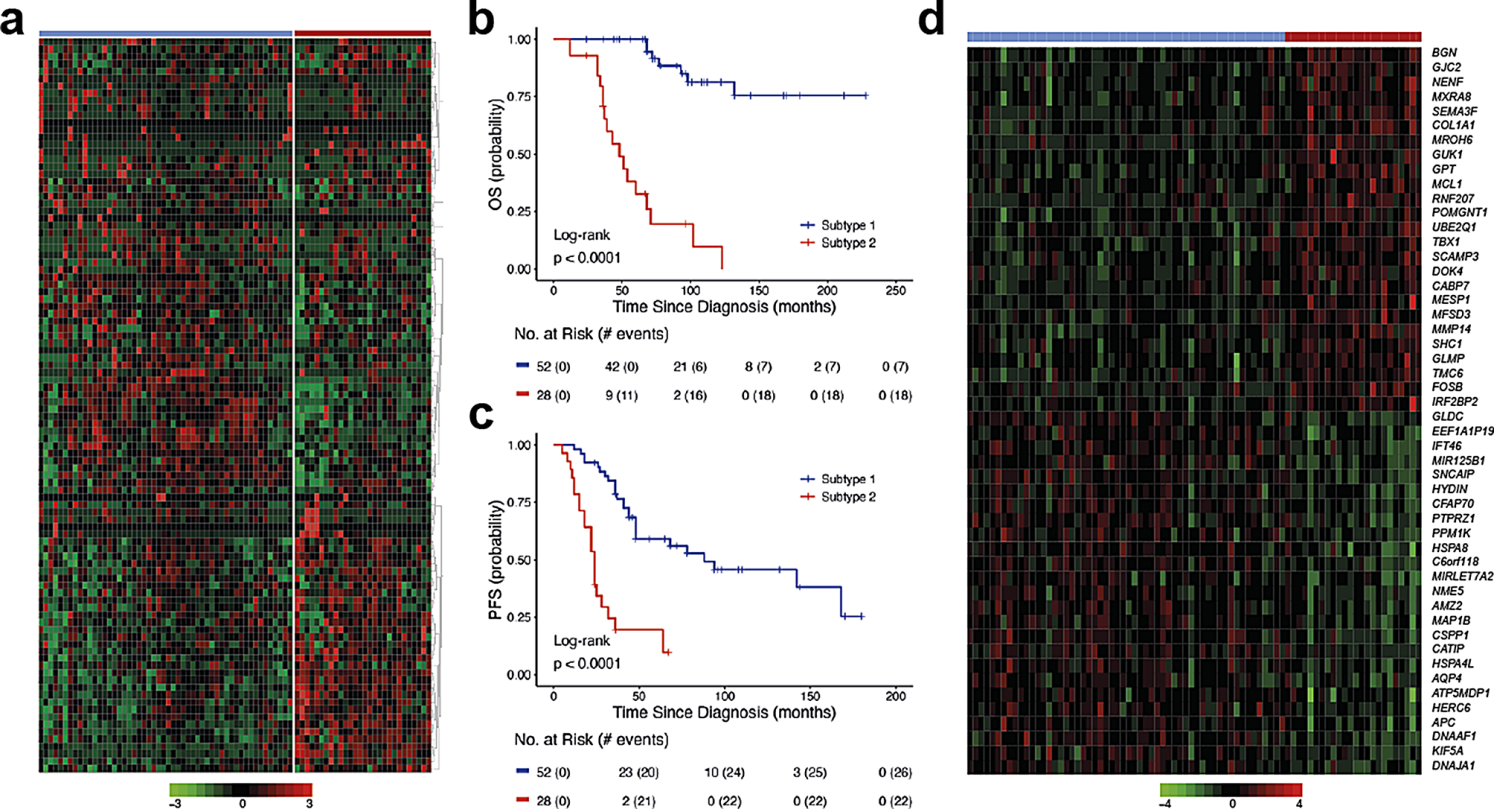
Supervised k-mean clustering of multigene survival data (**a**) defined a set of 100 genes (metagene set) that subdivided ST-EPN RELA into two transcriptome subtypes (TRS): favorable (*n* = 52) and unfavorable (*n* = 28). Two identified TRS were associated with patients’ OS (**b**) and PFS (**c**). **d**) Heatmap of top 20 most confident genes differentially expressed between clinically relevant TRS with BGN on the top of this list

Multiple gene OS analysis (see Methods) identified 1892 survival-associated genes with *BGN* on the top of the list (Suppl. Table 4). Among them, 253 genes disclosed independent hazard ratios (HR) by Cox regression analysis. In total, 1545 genes (147 with independent HR) were associated with favorable OS; among them, genes of the coiled-coil domain containing family (*CCDC*; *n* = 16), family with sequence similarity (*FAM*; *n* = 25), keratin family (*KRT*; *n* = 21), small nucleolar RNA family (*SNORD*; *n* = 15), and zinc finger protein family (*ZNF*; *n* = 23) prevailed. In contrast, 347 genes (106 with independent HR) were defined as unfavorable molecular indicators; among them, mitogen-activated protein kinase family (*MAPK*; *n* = 11), protocadherin family genes (*PCDH*; *n* = 11), and ribosomal protein family L/S (*RPL/RPS*; *n* = 11) were frequent. Moreover, 1423/1892 (75%) of these genes were also associated with PFS in ST-EPN ZFTA-RELA. Supervised k-mean clustering defined a set of 100 genes (metagene set) which subdivided ST-EPN ZFTA-RELA into two transcriptome subtypes with favorable/standard (*n* = 52; 5-year PFS – 50%; 5-year OS – 100%; 10-year OS – 75%) and unfavorable (*n* = 28; 5-year PFS – 10%; 5-year OS – 30%; 10-year OS − 0) clinical outcomes (Fig. 2a-c; Table 2). The favorable subtype was associated with fusion type 1 (45%), whereas the unfavorable subtype disclosed frequent 1q gain (60%), and fusion type 2 (45%) (Table 3). There were no associations between the prognostically relevant transcriptome subtypes and other clinical-molecular variables. DEG analysis identified 232 genes with *BGN* as the top-ranked gene within the unfavorable subtype and *INTU* – within the favorable subset (Fig. 2d; Suppl. Table 5). By gene ontology analysis, the favorable ST-EPN subtype was associated with cilium motility and assembly, axoneme, and cytoskeleton microtubule pathways, whereas the unfavorable – with the extracellular matrix, collagen metabolism, angiogenesis, and cell migration/motility, pathways (Suppl. Figure 1a; Suppl. Tables 6 and 7). Cell type-specific gene set expression analysis (GSEA) disclosed that the favorable subtype was enriched with transcriptome signatures of ciliated epithelial and neuroepithelial cells, human radial glial cells, and cortex embryonic astrocytes, whereas the unfavorable subtype was enriched in signatures of mesenchymal stromal cells, embryonic brain endothelial and microglial cells, and embryonic neural stem cells (Suppl. Table 8). By inspection of methylation level between favorable and unfavorable cases it was possible to identify *n* = 656 differential CpG sites (Suppl. Table 9), however overlap with detected DEGs locations was only 2%.

**Table 3:** Clinical and molecular variables for ST-EPN ZFTA-RELA relevant transcriptome subtypes

Variable	Favorable subtype (52)	Unfavorable subtype (28)	*P*-value
Age: Median; Children/Adult	10; 80%/20%	11; 85%/15%	NS
Gender: Male/Female	60%/40%	65%/35%	NS
M stage: M0/M2-3	95%/5%	95%/5%	NS
Removal: Gross total/Near total	50%/50%	55%/45%	NS
Radiotherapy: Local/Craniospinal	90%/10%	90%/10%	NS
Chemotherapy	55%	60%	NS
Recurrence	50%	75%	< 0.01
5-year PFS	60%	10%	< 0.01
Death	15%	60%	< 0.01
5-year OS	100%	35%	< 0.01
10-year OS	80%	10%	< 0.01
1q gain	25%	50%	< 0.01
9p loss	45%	60%	< 0.01
*CDKN2A/B* homo deletion	35%	35%	NS
22q loss	35%	25%	NS
Monosomy X	30%	30%	NS
Type *ZFTA-RELA* Fusion 1	45%	20%	< 0.01
Type *ZFTA-RELA* Fusion 2	10%	35%	< 0.01
Type *ZFTA-RELA* Fusion 3	15%	15%	NS
Type *ZFTA-RELA* Fusion 4	30%	30%	NS
ST-RELA-Variable Fraction	15%	35%	< 0.01
ST-G2M-Phase Fraction	5%	5%	NS
ST-S-Phase Fraction	5%	5%	NS
ST-Metabolic Fraction	8%	5%	NS
ST-Neuronal-Precursor-like Fraction	16%	15%	NS
ST-Radial-Glia-like Fraction	27%	12%	< 0.01
ST-Interferon-Response Fraction	12%	3%	< 0.01
ST-Ependymal-like Fraction	10%	5%	< 0.01
Normal Cell Fraction	5%	5%	NS

### Cell content differences in clinically relevant transcriptome ST-EPN ZFTA-RELA subtypes

**Fig. 3:**
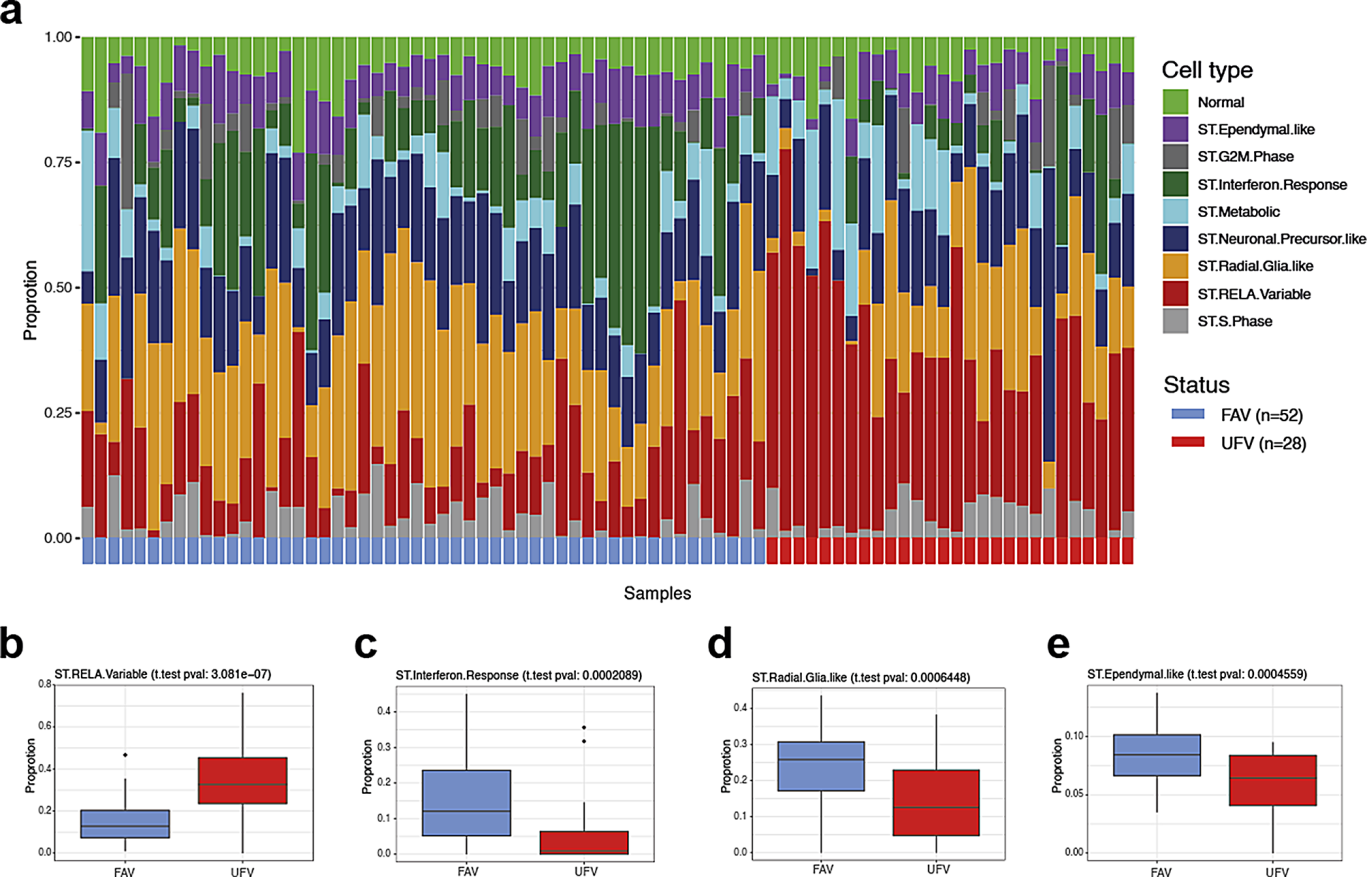
**a**) Bar plots of predicted relative proportions of EPN ZFTA cell types in bulk tumor gene expression profiles. Annotation provides favorable (FAV; blue) and unfavorable (UFV; red) status for each tumor sample. (**b**-**e**) Boxplots of statistically significant differences between EPN ZFTA favorable and unfavorable in proportions of ST-RELA-Variable (**b**), ST-Interferon-Response (**c**), ST-Radial-Glial (**d**) and ST-Ependymal (**e**) neoplastic cell subpopulations

We performed deconvolution analysis of bulk RNA-seq data to decipher ST-EPN ZFTA-RELA inter- and intra-tumoral cellular heterogeneity. For this purpose, we used a published single-cell RNA-seq dataset [14] that was composed of ST-EPN ZFTA-RELA (*n* = 5) covering 10 clusters of neoplastic cells that exhibited molecular signatures matching different transcriptome metaprograms (see below). Deconvolution analysis of bulk RNA profiles was performed using the BayesPrism computational program (see Methods), and significant proportions of neoplastic cells (more than 80%) were identified in all tumor samples. The proportion of non-tumoral cells was quite low (median ~ 7%). Based on the deconvolution analysis of single-cell molecular signatures, the bulk RNA-seq ST-EPN dataset was composed of two mitotic/proliferative cell programs (*ST-S-Phase* – 5% and *ST-G2/M-Phase* – 5%), two progenitor cell programs (*ST-Radial-Glia-Like* – 15% and *ST-Neuronal-Precursor-Like* – 15%), differentiated cell programs (*ST-Ependymal-Like* – 10%), interferon signaling program (*ST-Interferon-Response* – 10%), metabolic program (*ST-Metabolic* – 10%), and extracellular matrix program (*ST-RELA-Variable* – 20%) (Fig. 3a). A higher than median proportion of *ST-RELA-Variable* cell type subpopulation conferred the shortest OS (*p* < 0.01; Suppl. Figure 1b), whereas high *ST-Ependymal-Like* and *ST-Interferon-Response* cell fractions were associated with favorable clinical outcomes (*p* < 0.01 and *p* < 0.01 respectively; Suppl. Figure 1c, d). In addition, the shortest PFS but not OS was identified for higher *ST-Radial-Glia-Like* subpopulation (*p* < 0.01; not shown).

We further analyzed cell content within clinically relevant ST-EPN transcriptome subtypes (Fig. 3a-e; Table 3). In this analysis, the unfavorable subtype showed higher proportion of *ST-RELA-Variable* (35% vs. 15%; *p* < 0.01) cell subpopulation (Fig. 3b). In contrast, the clinically favorable ST-EPN subtype was composed of differentiated *ST-Interferon-Response* (12% vs. 3%; *p* < 0.01), *ST-Radial-Glia-Like* (27% vs. 12%; *p* < 0.01) and *ST-RELA-Ependymal-like* (10% vs. 5%; *p* < 0.01) cell subpopulations (Fig. 3c-e). There were no differences in other cell subpopulations between clinically relevant ST-EPN subtypes.

To verify the deconvolution results detected for the ST-EPN cohort, gene set variance analysis (GSVA) was performed as an alternative computational method on mean gene expression values computed from RPKM matrices generated for favorable and unfavorable transcriptome ST-EPN subsets, as described (see Methods). GSVA results showed the enrichment patterns in expression signatures of the identified neoplastic cell subpopulations within the clinically relevant transcriptome subtypes reflecting the results of bulk RNA deconvolution analysis for *ST-RELA-Variable*, *ST-Interferon-Response* and *ST-Radial-Glia-like* (Suppl. Figure 1e).

### *BGN* expression as a possible biomarker for ST-EPN ZFTA-RELA risk stratification

*BGN* was identified by multiple gene survival testing (see Methods) as a top gene associated with poor outcomes (HR 17.85 for PFS and 45.48 for OS; log-rank; p-value < 0.01; see. Suppl. Table 4; Suppl. Figure 2a, b), and was also significantly overexpressed in the unfavorable transcriptome ST-EPN subtype (Suppl. Figure 2c). This gene is known to be associated with maintenance of the extracellular matrix structure and located on chromosome X. Nevertheless, the overexpression of *BGN* in unfavorable subset was observed as significantly independent of patients’ sex (Suppl. Figure 2d, e). Across ST-EPN cell types *BGN* expression was found to be active across all tumor cell types, mostly enriched in *ST-RELA-Variable* and cell cycle-associated subpopulations, but almost not expressed in normal cells fraction (Suppl. Figure 2f). No significant difference in *BGN* expression was seen between ZFTA-RELA and ZFTA-non RELA ST-EPN (Suppl. Figure 2 g), but gene expression for ST-EPN ZFTA-RELA was significantly higher as compared to ST-EPN-YAP1 and ST-EPN-SE (Suppl. Figure 2 h).

There were no associations between *BGN* expression levels and DNA profiles at the gene location (Xq28). However, we identified a negative correlation between *BGN* expression and methylation levels of two CpG sites within the gene promoter region (cg21179255 and cg04177332; Suppl. Figure 3a, b). Moreover, low methylation levels for these two CpGs were associated with poor OS (log-rank; p-value = 0.01 and < 0.01 respectively) (Table 2; Suppl. Figure 3c, d). Nevertheless none of the CpGs lying within *BGN* loci were significantly differentially methylated between favorable and unfavorable ST EPN transcriptome subtypes (Suppl. Table 9), thus suggesting only an inverse correlation between gene expression levels and methylation of a few GpGs within the promoter region as association.

In a Cox regression model accounting for all clinical and molecular data, the unfavorable ST-EPN subtype and *BGN* expression were independently associated with poor survival (Table 2). Further, we compared stratification regression models with and without these independent variables. The inclusion of these molecular parameters significantly improved outcome prediction for the current ST-EPN cohort thus reducing prediction errors. Similar results were obtained when we compared receiver areas under curves (AUC) and operating characteristic curves (ROC) for the Cox models at different time points. Thus, the inclusion of the transcriptional subtype and/or *BGN* expression data resulted in the improvement of the ST-EPN risk stratification model.

In addition, survival analyses of public gene expression data generated with the Affymetrix platform for multi-institutional extended ST-EPN cohort [27] also showed unfavorable outcomes for tumors with high *BGN* expression, thus confirming data obtained with our RNA sequencing analysis (Suppl. Figure 3e).

### IHC with biglycan a possible tool for ST-EPN ZFTA-RELA prognostication

We applied a biglycan/BGN antibody (see Methods) to stain 70 samples with accessible tumor sections from the current transcriptome analysis cohort (screening set) and 56 samples from an independent molecularly diagnosed ST-EPN ZFTA-RELA cohort applied in previous studies (validation set) [27, 37].

**Fig. 4:**
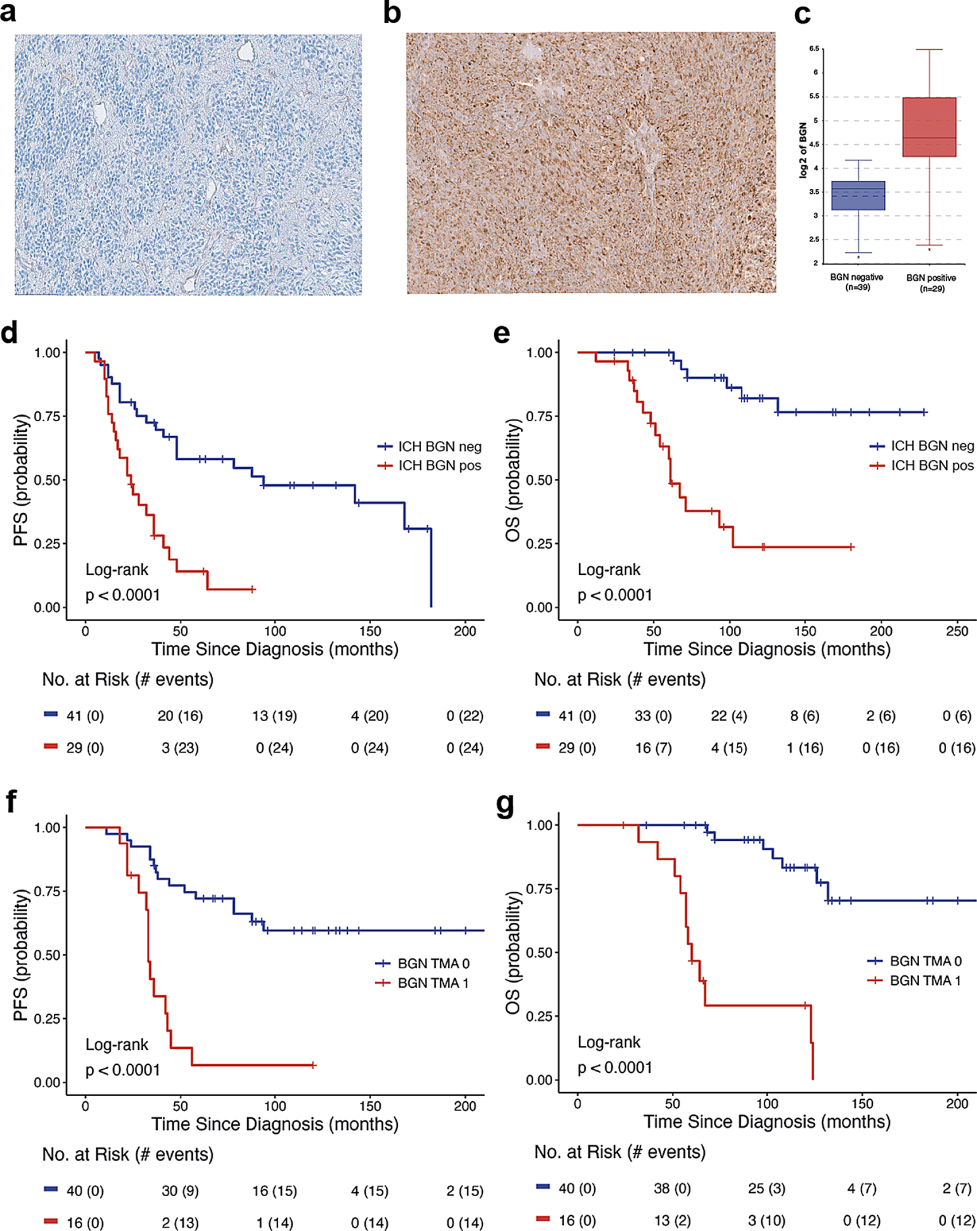
Two variants of BGN protein immunostaining were detected: (**a**) Negative - expression in the tumor vessels predominantly. (**b**) Positive - intense dot-like BGN expression in tumor cells. BGN expression levels were significantly higher in immunopositive ST-EPN RELA (**c**). Survival analysis revealed that BGN immunopositivity is significantly associated with worse clinical outcomes in both the screening (**d**,**e**) and validation (**f**,**g**) sets of ST-EPN RELA

The two following patterns of BGN immunostaining were detected: (i) Expression was found in the tumor vessels (including microvascular proliferates), and patched collections of tumor cells (*n* = 41 in the screening set and *n* = 40 in the validation set; Fig. 4a). These samples were considered BGN-negative. (ii) Diffusely and predominantly dot-like immunostaining throughout the entire tumor (*n* = 29 in the screening set and *n* = 16 in the validation set; Fig. 4b). These samples were designated as BGN-positive. Two investigators showed perfect interobserver agreement for this categorization (κ = 1), and we did not find differences in terms of staining intensity across both tumor sets. In addition, 6/16 (40%) ST-EPN with ZFTA*-*non-RELA fusions were BGN-positive, whereas all ST-EPN-YAP1 studied (*n* = 18) were BGN-negative.

In the screening set, BGN expression data coincided strongly between mRNA and protein levels (correlation coefficient *r* = 0.857; *p* < 0.01; Fig. 4c). In addition, 90% ST-EPN with elevated *BGN* expression (*log2* > 4) were BGN-positive in contrast to 5% samples with low gene expression (*p* < 0.01). Also, most of the ST-EPN (86%) from the unfavorable transcriptome subtype were BGN-positive as compared to 12% ST-EPN allocated to the favorable subtype (*p* < 0.01).

Survival analysis revealed that BGN-positivity is significantly associated with worse clinical outcomes in both the screening (5-year PFS – 15% and 5-year OS – 45%) and validation (5-year PFS – 10% and 5-year OS – 40%) sets (Fig. 4d-g). Thus, the results of BGN IHC prognostic evaluation correlated closely with the survival data obtained by transcriptome analysis.

## Discussion

In this study, we aimed to investigate the molecular heterogeneity of retrospective analyzed ST-EPN ZFTA-RELA series and to identify reliable genomic marker(s) that can be applied with inexpensive, accessible, and efficient method(s) for the identification of possible ST-EPN risk categories. The clinical and molecular parameters, including CNVs and ZFTA_RELA fusions variants, were not identified as related to survival in this ST-EPN cohort. Thus, in contrary to previous studies [5, 30] homozygous *CDKN2A/B* deletion was not associated with ST-EPN survival that could be partly explained by differences in the number of patients included and variability in the treatment protocols.

In the current study, we focused on the prognostic evaluation of ST-EPN ZFTA-RELA transcriptome profiles generated with RNA sequencing. Thus, multiple gene survival analysis identified a set of survival-associated genes that were identified as strong predictors of tumor clinical behavior. In turn, k-mean clustering defined a metagene set that subdivided ST-EPN ZFTA-RELA into two transcriptome subtypes with contrary clinical outcomes. Gene ontology and deconvolution analyses identified that these subtypes were associated with specific signaling pathways and cellular composition that could underlie the ST-EPN clinical-molecular diversity. Thus, the favorable subtype was associated mostly with cilia/axoneme pathways and cell subpopulations with ependymal differentiation, radial-glial cells and interferon-response cell fractions. A previous study based on single-cell sequence analysis also showed that ST-EPN with “ependymal” transcriptome signatures showed favorable outcomes [14]. In contrast, the unfavorable transcriptome subtype was enriched with pathways and cell subpopulations associated with the extracellular matrix, angiogenesis, and cell motility thus suggesting their biological aggressiveness. Notably, single-cell sequencing identified mesenchymal EPN cell population in recurrent EPN thus stressing its possible association with tumor progression and suggesting acquired epithelial-mesenchymal transition [3, 10, 13].

*BGN* encodes biglycan protein (BGN), a key member of the small leucine-rich proteoglycan family, which is an important component of the extracellular matrix [8, 36]. Overexpression of *BGN* at mRNA and/or protein level has been associated with advanced tumor stages, metastases development, drug resistance, and poor prognosis in patients with ovarian, prostate, oral, colon, and gastric cancers [11, 16, 21, 22, 36]. In the current study, *BGN* was identified as a provisional biomarker of the ST-EPN “mesenchymal-like” unfavorable subtype and, also, as a strong prognostic indicator, confirmed in independent validation series. The clinically relevant *BGN* transcriptional diversity is associated with methylation within the gene promoter region and, respectively, might be driven by molecular mechanisms associated with epigenetic dysregulation.

Because *BGN* expression was an independent indicator of ST-EPN ZFTA-RELA’s poor prognosis, a risk stratification model including this “mesenchymal-associated” molecular pattern may act as a useful tool for further routine application. Moreover, *BGN* expression has potential usefulness for the development of ST-EPN therapy because inhibition of renal cell carcinoma growth has been promoted by biglycan siRNA-containing nanodevices in vivo models [22]. Risk stratification and accurate outcome prediction of future ST-EPN ZFTA-RELA cohorts in the absence of high-throughput profiling techniques may be enhanced by assessing *BGN* expression in routine neuropathology. For example, single gene RQ-PCR quantification, Taqman low-density arrays, or Nanostring-based analyses evaluating the expression of this gene might be easily developed in neuropathological practice after the elaboration of optimal cut-off levels for each method applied [5, 7, 17]. In addition, BGN protein expression was defined here as a prognostic indicator and its IHC may also be considered a potent marker for further ST-EPN ZFTA-RELA stratification. Moreover, because BGN immunopositivity was not identified in ST-EPN YAP1, the utility of this marker combined with L1CAM/ p65-RelA [12, 26] may be applied for diagnostic purposes in neuropathological settings.

## Conclusions

In summary, the ST-EPN molecular variant designated *ZFTA-RELA* exhibits clinically relevant transcriptional heterogeneity subdividing these tumors into two clear-cut molecular subsets: prognostically favorable and clinically aggressive, “mesenchymal-like” ST-EPN RELA respectively. Current results also indicate that integrating *BGN* expression in risk stratification models may improve ST-EPN ZFTA-RELA outcome prediction. It has important clinical relevance, as a simple expression analysis for this predictive molecular marker at the mRNA or protein level could be adopted in neuropathology laboratories worldwide, including low- and middle-income countries. Thus, rapid BGN-based risk stratification of ST-EPN RELA could help in assigning these patients to individual treatment protocols and future research should aim at validating the relevance of the proposed ST-EPN RELA stratification in prospective clinical trials.

## Electronic supplementary material

Below is the link to the electronic supplementary material.

Supplementary Material 1

Supplementary Material 2

## Data Availability

The RNA-seq dataset generated and analyzed in the current study (normalized gene expression counts matrix) with detailed annotation is available in the R2 platform (http://r2.amc.nl) under the name “Tumor Ependymoma FFPE - Korshunov - 80 - RPKM - epffpe”. The methylation data available in GEO database under access number GSE65362.

## Abbreviations

EPN: Ependymoma
ST: Supratentorial
PF: Posterior fossa
SP: Spinal
BGN: Biglycan
RT: Radiotherapy
CHT: Chemotherapy
DEG: Differentially expressed genes
CNV: Copy number variants
GSEA: Cell type-specific gene set expression analysis
GSVA: Gene set variance analysis
HR: Hazard ratio
PFS: Progression-free survival
OS: Overall survival
IHC: Immunohistochemistry
AUC: Areas under curves
