# Correction: Biglycan-driven risk stratification in ZFTA-RELA fusion supratentorial ependymomas through transcriptome profiling

**DOI:** 10.1186/s40478-025-02094-w

**Published:** 2025-08-21

**Authors:** Konstantin Okonechnikov, David R. Ghasemi, Daniel Schrimpf, Svenja Tonn, Martin Mynarek, Jan Koster, Till Milde, Tuyu Zheng, Philipp Sievers, Felix Sahm, David T.W. Jones, Andreas von Deimling, Stefan M. Pfister, Marcel Kool, Kristian W. Pajtler, Andrey Korshunov

**Affiliations:** 1 Hopp Children’s Cancer Center Heidelberg (KiTZ), Heidelberg, Germany; 2 Division of Pediatric Neuro-Oncology (B062), German Cancer Research Center (DKFZ) and German Cancer Consortium (DKTK), Heidelberg, Germany; 3 Pediatric Hematology and Oncology, University Medical Center Hamburg-Eppendorf, Hamburg, Germany; 4 Mildred Scheel Cancer Career Center HaTriCS4, University Medical Center Hamburg-Eppendorf, Hamburg, Germany; 5 Research Institute Children’s Cancer Center, Hamburg, Germany; 6 Department of Pediatric Hematology and Oncology, Heidelberg University Hospital, Heidelberg, Germany; 7 Clinical Cooperation Unit Neuropathology (B300), German Cancer Research Center (DKFZ), German Cancer Consortium (DKTK), National Center for Tumor Diseases (NCT), Heidelberg, Germany; 8 Department of Neuropathology, Heidelberg University Hospital, Heidelberg, Germany; 9 Center for Experimental and Molecular Medicine, Amsterdam University Medical Centers, University of Amsterdam and Cancer Center Amsterdam, Amsterdam, The Netherlands; 10 Clinical Cooperation Unit Pediatric Oncology, German Cancer Research Center (DKFZ) and German Consortium for Translational Cancer Research (DKTK), Heidelberg, Germany; 11 National Center for Tumor Diseases (NCT), Heidelberg, Germany; 12 Division of Pediatric Glioma Research (B360), German Cancer Research Center (DKFZ), Heidelberg, Germany; 13 Princess Máxima Center for Pediatric Oncology, Utrecht, 3584CS The Netherlands; 14 Clinical Cooperation Unit Neuropathology (B300), German Cancer Research Center (DKFZ), Im Neuenheimer Feld 280, 69120 Heidelberg, Germany

**Acta Neuropathologica Communications (2024) 13:4**

10.1186/s40478-024-01921-w.

In this article [1], Fig. 1 appeared incorrectly and have now been corrected in the original publication. For completeness and transparency, the old incorrect versions are displayed below.

Incorrect Fig. 1.

**Fig. 1:**
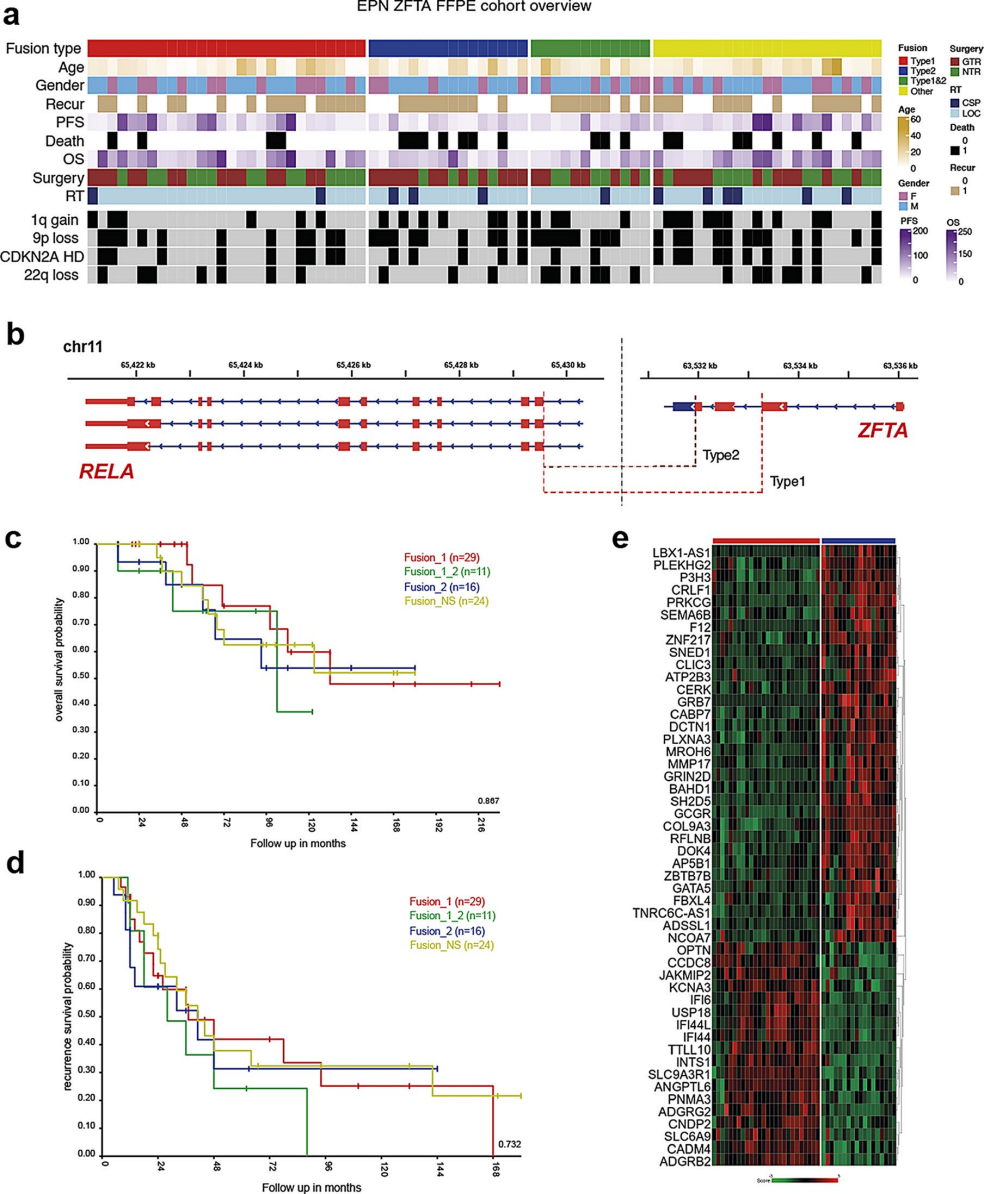
(**a**) Annotation onco-plot describing patient histological and molecular characteristics for target ZFTA-RELA ST-EPN tumors with available RNA sequencing data (*n* = 80). The following abbreviations were used: RT - radiotherapy, LOC - conformal local, CSP - craniospinal, PFS—progression-free survival, CNV—copy number variants. (**b**) Genomic locations the ZFTA-RELA fusion breakpoints stating the main types of the fusion. **c**, **d**) No survival differences were identified between the various ZFTA-RELA fusion types. **d**) Heatmap of significant differentially expressed genes between ZFTA-RELA fusion type 1 (*n* = 29) and 2 (*n* = 16)

Correct Fig. 1.

**Fig. 1:**
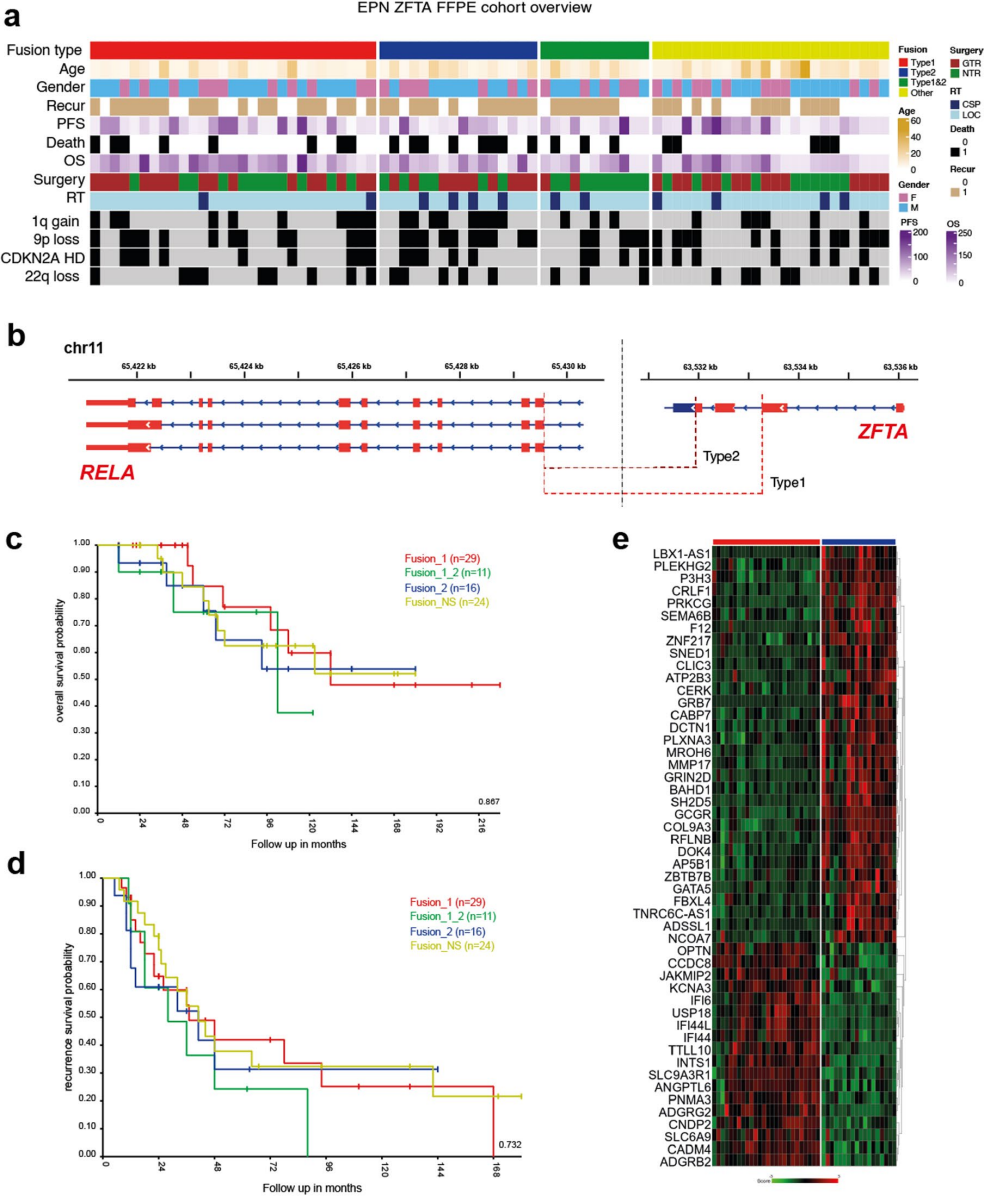
(**a**) Annotation onco-plot describing patient histological and molecular characteristics for target ZFTA-RELA ST-EPN tumors with available RNA sequencing data (*n* = 80). The following abbreviations were used: RT - radiotherapy, LOC - conformal local, CSP - craniospinal, PFS—progression-free survival, CNV—copy number variants. (**b**) Genomic locations the ZFTA-RELA fusion breakpoints stating the main types of the fusion. **c**, **d**) No survival differences were identified between the various ZFTA-RELA fusion types. **d**) Heatmap of significant differentially expressed genes between ZFTA-RELA fusion type 1 (*n* = 29) and 2 (*n* = 16)
