# Supplementary material for: Biglycan-driven risk stratification in ZFTA-RELA fusion supratentorial ependymomas through transcriptome profiling
Source: Acta Neuropathol Commun. 2025 Jan 7;13:4. doi: 10.1186/s40478-024-01921-w (PMC11706152; doi:10.1186/s40478-024-01921-w)
Supplement: Supplementary file 2 — Supplementary Material 2 [file 40478_2024_1921_MOESM2_ESM.docx]

**SUPPLMENTARY FIGURES**

**Supplementary Figure 1**

| 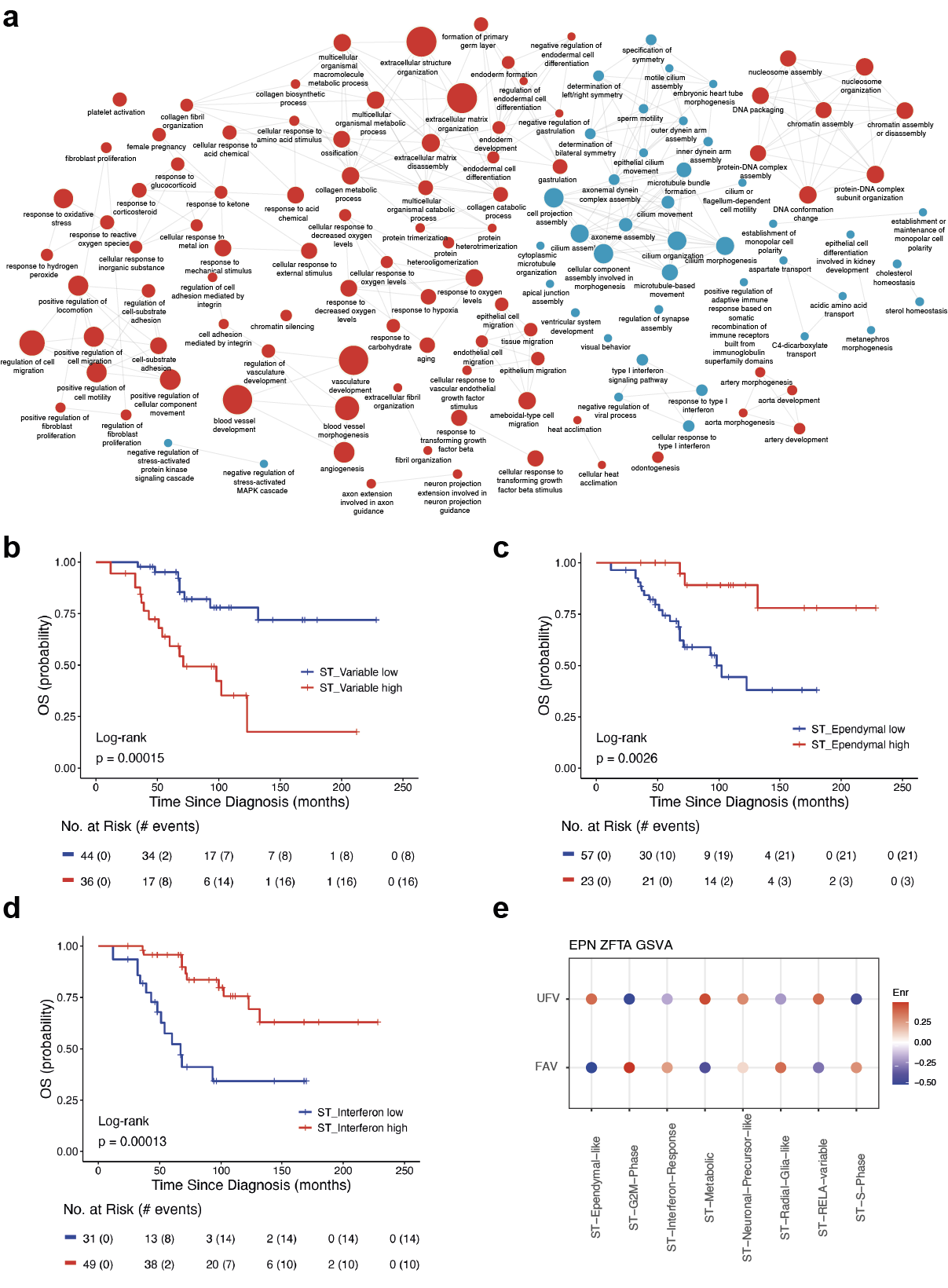 |
| --- |

**Suppl. Figure 1.** a) Visualization of gene ontology analysis results for differentially expressed genes between favorable (blue) and unfavorable (red) EPN ZFTA tumors. Thus, favorable subtype was associated with cilium motility, axoneme, and microtubule pathways, whereas the unfavorable – with the extracellular matrix, angiogenesis, and cell migration pathways. Kaplan-Meyer overall survival curves with the impact of "low" and "high" ST-RELA-Variable (b), ST-Ependymal (c), and ST-Interferon-Response (d) cell types which revealed prognostic significance across tumor cohort. e) GSVA analysis confirmed the results of bulk RNA deconvolution for ST-RELA-Variable, ST-Interferon-Response and ST-Radial-Glia-like cell subpopulations.

**Supplementary Figure 2**

| 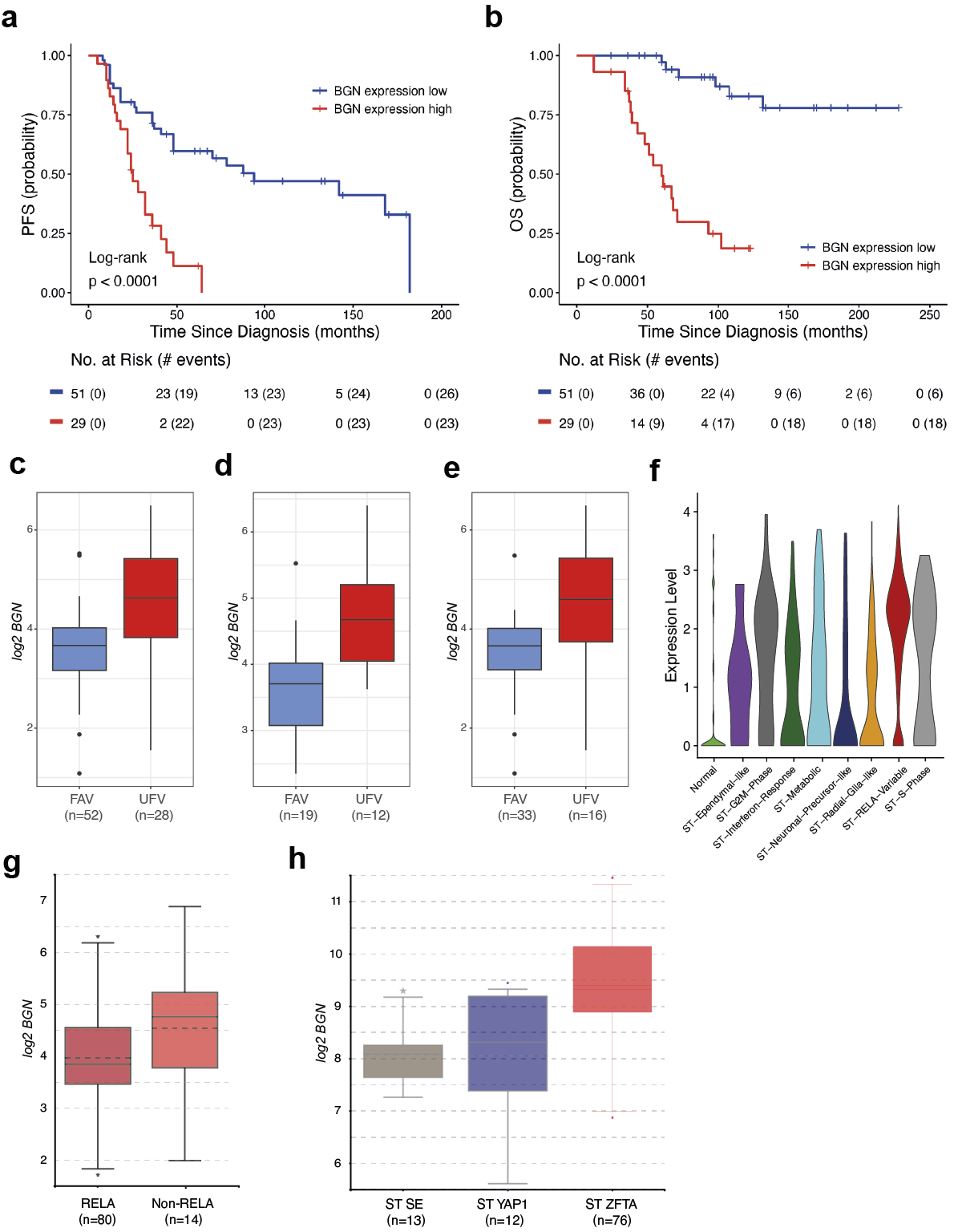 |
| --- |

**Suppl. Figure 2.** High *BGN* expression was associated with unfavorable PFS (a) and OS (b) as compared to low expression levels. *BGN* expression levels were significantly higher in unfavorable transcriptome subtype (c), this trend was stable for female (d) and male (e) patients analyzed separately. *BGN* expression was enriched mostly in ST-RELA-Variable and cell cycle-associated subpopulations, but almost not expressed in normal cells fraction (f). No significant difference in *BGN* expression was seen between ST-EPN RELA and ST-EPN ZFTA-non RELA (g) but was significantly higher as compared to ST-SE and ST-EPN with *YAP1_MAMLD1* fusion (h).

**Supplementary Figure 3.**

| 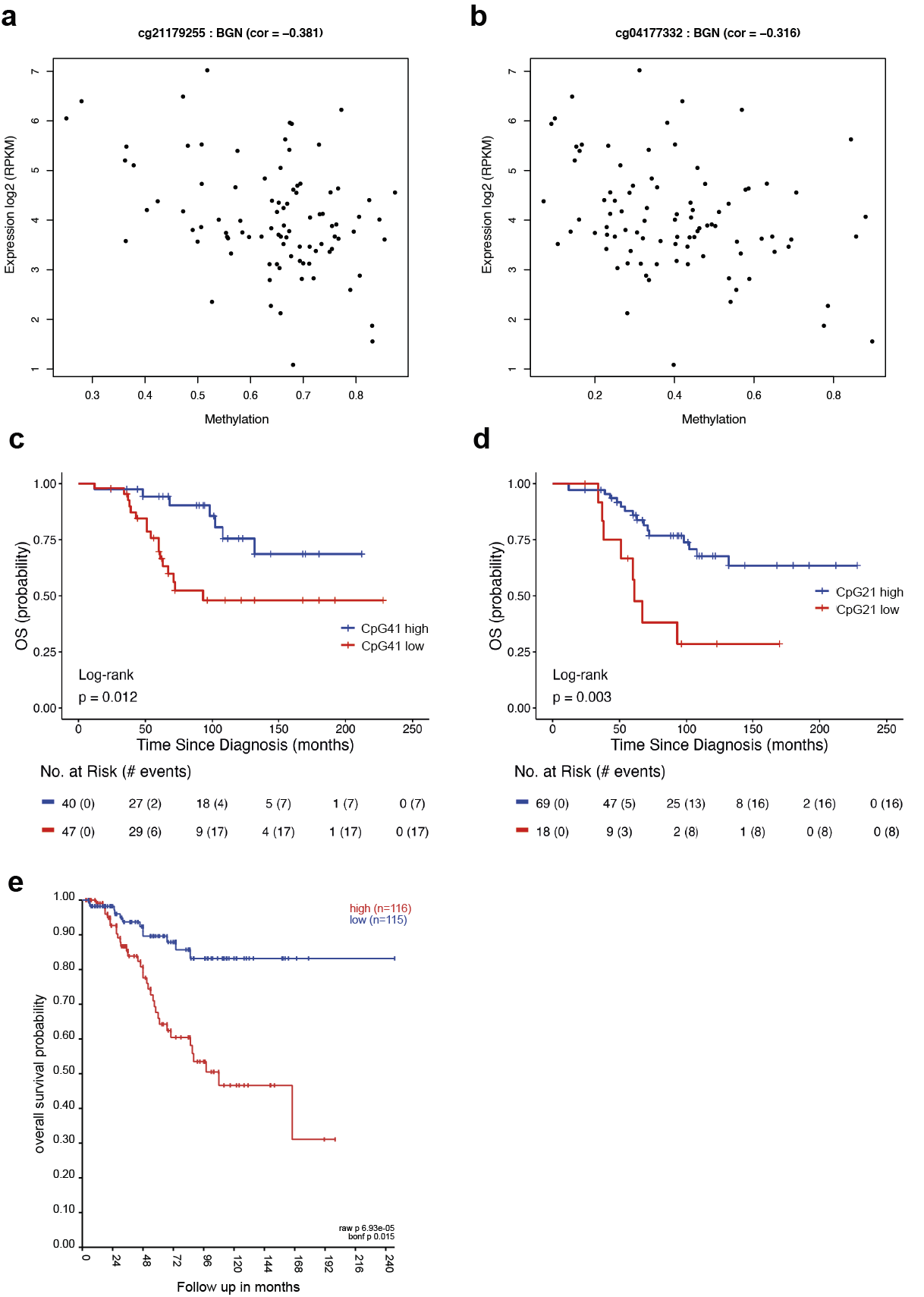 |
| --- |

**Suppl. Figure 3.** Comparing *BGN* expression and epigenetic data overlapping gene promoter region (2500 upstream), methylation levels of two CpG sites - cg04177332 (a) and cg21179255 (b) were significantly lower in ST-EPN RELA samples with high *BGN* expression and *vice versa*. (c,d). Low methylation levels for these two CpGs (with a cut-off beta-value level = 0.40 for cg04177332 and 0.55 for cg21179255) were associated with unfavorable OS. (e). Survival analysis of public gene expression data generated with the Affymetrix platform for independent multi-institutional ST-EPN cohort showed unfavorable outcomes for tumors with high *BGN* expression levels using a Bonferroni correction for multiple testing, thus confirming data obtained with RNA sequencing data analysis.

**SUPPLEMENTARY TABLES**

**Suppl. Table 1.** Clinical data and cohort overview.

**Suppl.** **Table 2.** Differentially expressed genes between ST-EPN-ZFTA fusion type1 and type 2. Min corrected p-value 0.01.

**Suppl.** **Table 3.** Gene ontology analysis of differentially expressed genes between ST-EPN-ZFTA fusion type1 and type 2.

**Suppl.** **Table 4.** Significant survival associated genes within ST-EPN-ZFTA tumors.

**Suppl.** **Table 5.** Differentially expressed genes between favourable and unfavourable ST-EPN-ZFTA subgroups. Min corrected p-value 0.01.

**Suppl.** **Table 6.** Gene ontology analysis of differentially expressed genes specific for favourable ST-EPN-ZFTA cases.

**Suppl.** **Table 7.** Gene ontology analysis of differentially expressed genes specific for unfavourable ST-EPN-ZFTA cases.

**Suppl.** **Table 8.** Cell type signature inspection of differentially expressed genes between ST-EPN-ZFTA favourable and unfavourable cases.

**Suppl.** **Table 9.** Differentially methylated cites genes between ST-EPN-ZFTA favourable and unfavourable cases. Min adjusted p-value (q-value) 0.05.
